# Burden of Self-reported Acute Gastrointestinal Illness in Cuba

**DOI:** 10.3329/jhpn.v27i3.3377

**Published:** 2009-06

**Authors:** Pablo Aguiar Prieto, Rita L. Finley, P.K. Muchaal, Michele T. Guerin, Sandy Isaacs, Arnaldo Castro Domínguez, Gisele Coutín Marie, Enrique Perez

**Affiliations:** ^1^ National Environmental Health Unit, Ministry of Health, Ciudad de La Habana, CP 10 400, Cuba; ^2^ Centre for Food-borne, Environmental and Zoonotic Infectious Diseases, Public Health Agency of Canada, Guelph, Ontario, N1H 8J1, Canada; ^3^ Department of Population Medicine, Ontario Veterinary College, University of Guelph, Guelph, Ontario, N1G 2W1, Canada; ^4^ Area of Health Surveillance and Disease Management, Food Safety Group, Pan American Health Organization, Rio de Janeiro, 25040-004, Brazil

**Keywords:** Cross-sectional studies, Developing countries, Diarrhoea, Epidemiology, Public health, Retrospective studies, Risk factors, Seasonal variation, Sentinel sites, Cuba

## Abstract

Acute gastrointestinal illness is an important public-health issue worldwide. Burden-of-illness studies have not previously been conducted in Cuba. The objective of the study was to determine the magnitude, distribution, and burden of self-reported acute gastrointestinal illness in Cuba. A retrospective, cross-sectional survey was conducted in three sentinel sites during June-July 2005 (rainy season) and during November 2005–January 2006 (dry season). Households were randomly selected from a list maintained by the medical offices in each site. One individual per household was selected to complete a questionnaire in a face-to-face interview. The case definition was three or more bouts of loose stools in a 24-hour period within the last 30 days. In total, 97.3% of 6,576 interviews were completed. The overall prevalence of acute gastrointestinal illness was 10.6%. The risk of acute gastrointestinal illness was higher during the rainy season (odds ratio [OR]=3.85, 95% confidence interval [CI] 3.18-4.66) in children (OR=3.12, 95% CI 2.24-4.36) and teens (OR=2.27, 95% CI 1.51-3.41) compared to people aged 25-54 years, in males (OR=1.24, 95% CI 1.04-1.47), and in the municipality of Santiago de Cuba (OR=1.33, 95% CI 1.11-1.61). Of 680 cases, 17.1-38.1% visited a physician, depending on sentinel site. Of the cases who visited a physician, 33.3-53.9% were requested to submit a stool sample, and of those, 72.7-100.0% complied. Of the cases who sought medical care, 16.7- 61.5% and 0-31.6% were treated with antidiarrhoeals and antibiotics respectively. Acute gastrointestinal illness represented a substantial burden of health compared to developed countries. Targeting the identified risk factors when allocating resources for education, food safety, and infrastructure might lower the morbidity associated with acute gastrointestinal illness.

## INTRODUCTION

Acute gastrointestinal illness is an important public-health issue worldwide. In developed countries, estimates of monthly prevalence range from 4.5% to 11% (1-9). Although illness is typically mild and self-limiting, acute gastrointestinal illness imposes a substantial economic burden to the population and healthcare system (3-5,9). Diarrhoea is one of the primary causes of morbidity and mortality among children aged less than five years in the developing world; globally, it is estimated that there are 3.2 episodes of diarrhoea per child-year and 4.9 deaths per 1,000 children per year due to diarrhoeal illness (10).

The Pan American Health Organization (PAHO) supports health needs and initiatives within the Americas, with a focus on the Latin American and Caribbean countries. Within an initiative sponsored by the World Health Organization (WHO), the PAHO and the Centre for Food-borne, Environmental and Zoonotic Infectious Diseases of the Public Health Agency of Canada worked jointly with the Cuban Ministry of Health to develop a study aimed at understanding the burden of illness associated with gastrointestinal diseases in Cuba and how it compares with other countries.

Our objective was to determine the temporal and demographic distribution and burden of self-reported acute gastrointestinal illness in Cuba. It is anticipated that this information will assist the Ministry of Health in assigning resources for education and food safety.

## MATERIALS AND METHODS

### Study design

A cross-sectional survey was conducted within three purposively-selected sentinel sites in Cuba (Fig.). The main municipality within provinces representing differences in urban-rural mix, from different regions of the country, and the differences in their predefined risk rating for acute gastrointestinal illness were selected. The sentinel sites were: (a) municipality of Cienfuegos (CF; provincial capital, 333 sq km, population–164,180) within the province of Cienfuegos, (b) municipality of Santiago de Cuba (SC; provincial capital, 1,024 sq km, population–494,915) within the province of Santiago de Cuba, and (c) municipality of Centro Habana (CH; 4 sq km, population–157,539) within the province of Ciudad de La Habana (11). The sentinel sites represent ∼7.3% of the total Cuban population (11,241,291) in 2004 (12).

**Fig.**
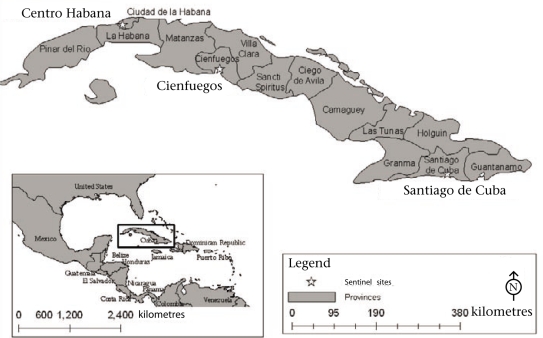
Location of sentinel sites in Cuba

The sampling frame consisted of a list of households served by the family doctors' offices within each sentinel site. Five households from each office were randomly selected for inclusion. Trained nurses or hygiene and epidemiology technicians associated with each medical office administered face-to-face interviews. One individual per household was selected to participate in the interview. It was our intention to interview the household member whose birthday was the closest to the date of interview. However, in most instances, the household member who answered the door was interviewed instead of having the interviewer return at a later date to implement the study on the appropriate individual. For logistical reasons, we were unable to carry out follow-ups. This resulted in a non-random selection of participants, biasing towards women aged 25-54 years. If the individual met the exclusion criteria (outlined below), another person from the household was selected. Participants aged ≥18 years read and signed an informed consent record for themselves or for an individual they were a proxy for. Proxy respondents were accepted for individuals aged less than 15 years. Parents or guardians of participants aged 15-17 years were given the option of having the youth respond directly to the survey. All interviews were conducted in Spanish. An identification number was assigned to each respondent to ensure confidentiality.

Seasonal variation in the incidence of acute gastrointestinal illness has previously been identified from surveillance data in Cuba (13). Therefore, interviews were administered during two different time periods: June and July 2005 (representing the rainy season) and November 2005 to January 2006 (representing the dry season). A separate group of participants was selected for each season.

The target sample size of 3,288 interviews per season was calculated based on a confidence of 95%, a precision of 1.5%, and an estimated monthly incidence of 0.0208 (CF), 0.0533 (SC), and 0.0375 (CH). The latter was derived from Cuban statistics on the number of medical visits for diarrhoea and data obtained from hidden morbidity surveys conducted in the provinces (CF, 25% hidden morbidity; SC, 64%; CH, 45%). Within neighbourhoods, households are assigned to a doctor's office. Therefore, the original sample size was then increased to adjust for clustering of households within a family doctor's office; a conservative estimate of ρ=0.2 was used.

Data were collected using a questionnaire developed through the modification of a pre-existing population-based burden-of-disease questionnaire (14); questions were amended or added after the Cuban Ministry of Health officials and provincial epidemiologists provided input on exposures hypothesized to be associated with acute gastrointestinal illness in Cuba. The questionnaire was pretested in CH on a sample of the population, using one-on-one interviews, by personnel trained on the illness characteristics, survey methodology, and professional ethics. The questionnaire was further refined prior to the implementation of the study.

We defined acute gastrointestinal illness as three or more bouts of loose stools in a 24-hour period. Respondents were identified as cases if they experienced acute gastrointestinal illness within the last 30 days. The exclusion criteria included individuals who had been diagnosed with chronic gastrointestinal illness, e.g. irritable bowel syndrome or Crohn's disease, by a physician, or who associated their illness with pregnancy. Cases were also asked about additional symptoms, duration of illness, illness in other household members, physician-visits, laboratory tests requested by their physician, compliance with testing, and any treatments received.

### Analysis of data

All sets of questionnaire were reviewed for legibili-ty and completeness. Data were entered into the Epi Info software (version 5.1) (Centers for Disease Control and Prevention, Atlanta, GA, USA) creating one database per sentinel site per season. Entered data were visually examined for accuracy against each questionnaire.

Data were analyzed using the SAS software (version 9.2) (SAS Institute Inc., Cary, NC, USA). Demographic characteristics were compared with the 2004 Census data (11) to determine how representative the study population was of the target population. Preliminary analysis of data included testing the association between acute gastrointestinal illness and each demographic factor using univariable logistic regression. The category with the highest number of observations was used as the reference group for each variable. Variables with p≤0.05 (Wald's test, two-tailed) were considered significant. We then offered season, sentinel site, gender, and age to a multivariable logistic-regression model. Occupation and education were not offered to the multivariable model due to correlation between one or more categories of these variables and the gender and age variables. For example, there was high correlation (Kendall's τ _b_=0.72, p<0.001) for occupation (≤17 years old, i.e. not old enough to work, and the age [0-12 year(s)] and between occupation (housewife) and gender (female) (τ=0.41, p<0.001). To investigate more fully the differences within seasons and sentinel sites, all additional analyses were conducted on stratified data. That is, separate estimates were calculated for each season and for each season within each sentinel site.

The primary outcome was the monthly prevalence of acute gastrointestinal illness. This was calculated as the number of respondents reporting acute gastrointestinal illness in the 30 days prior to the interview divided by the total number of respondents. Age-specific and gender-specific risks of morbidity for each stratum were calculated as the number of ill respondents in each category divided by the total number of respondents in the same category, multiplied by 100; confidence intervals for risks of morbidity were calculated using the OpenEpi software (version 2.2) (15). Multivariable logistic regression models were used for examining gender and age as risk factors for acute gastrointestinal illness. Both the variables were forced into the models regardless of statistical significance so that parame-ter estimates for gender were adjusted for age and vice-versa. Using odds ratios to approximate risk ratios, the risk differences were used in determining if age-specific risks were the same for each sentinel site within a season. The sentinel sites were also compared within seasons using the risk differences. Descriptive statistics for characteristics of illness, healthcare, and burden of illness included frequencies and proportions for categorical variables, and means and standard deviations, medians, and maximums or ranges for quantitative variables. Means were compared using *t*-tests; comparisons were made between sentinel sites within a season.

## RESULTS

The results of the preliminary analysis of data confirmed our a priori hypothesis of seasonal variation and differences in risk between sentinel sites (Table 1).

**Table 1.** Distribution of demographic characteristics of survey respondents, and univariable and multivariable logistic regression models examining demographic characteristics associated with acute gastrointestinal illness in Cuba (n=6,399)

Variable	Frequency (%)	Univariate		Multivariable	
		OR (95% CI)	p value	OR (95% CI)	p value
Season					
Dry	3,187 (49.8)	0.3 (0.2-0.3)	<0.001	0.3 (0.2-0.3)	<0.001
Rainy	3,212 (50.2)	Ref	-	Ref	-
Sentinel site			0.143		0.009
Cienfuegos	1,096 (17.1)	0.9 (0.8-1.2)	0.561	0.9 (0.7-1.2)	0.584
Centro Habana	2,193 (34.3)	0.8 (0.7-0.9)	0.049	0.7 (0.6-1.0)	0.003
Santiago de Cuba	3,110 (48.6)	Ref	-	Ref	-
Gender					
Male	2,044 (31.9)	1.2 (1.0-1.4)	0.020	1.2 (1.0-1.5)	0.015
Female	4,355 (68.1)	Ref	-	Ref	-
Age-group (years)			<0.001		<0.001
0-12	201 (3.1)	4.1 (2.9-5.6)	<0.001	3.1 (2.2-4.4)	<0.001
13-17	175 (2.7)	2.2 (1.5-3.3)	<0.001	2.3 (1.5-3.4)	<0.001
18-24	563 (8.8)	1.0 (0.7-1.4)	0.969	1.1 (0.8-1.5)	0.664
25-54	3,214 (50.2)	Ref	-	Ref	-
55-64	1,046 (16.4)	0.9 (0.7-1.1)	0.255	0.8 (0.7-1.1)	0.173
65+	1,200 (18.8)	1.0 (0.8-1.3)	0.982	1.0 (0.8-1.3)	0.886
Occupation			<0.001	‡	
Labourer	687 (10.7)	0.9 (0.6-1.2)	0.324		
Services	594 (9.3)	1.4 (1.1-1.9)	0.020		
Administration	204 (3.2)	1.8 (1.2-2.7)	0.008		
Director	37 (0.6)	1.5 (0.6-3.9)	0.385		
Student	391 (6.1)	0.8 (0.5-1.2)	0.318		
Professional	670 (10.5)	0.9 (0.7-1.3)	0.808		
Retired	1,271 (19.9)	1.1 (0.8-1.3)	0.710		
Housewife	1,725 (26.9)	Ref	-		
Self-employed	171 (2.7)	1.0 (0.6-1.7)	0.972		
Unemployed	170 (2.7)	1.1 (0.6-1.8)	0.757		
Other	87 (1.4)	1.4 (0.7-2.7)	0.297		
≤17 years old (too young to work)	376 (5.9)	3.4 (2.5-4.5)	<0.001		
No response∗	16 (0.3)				
Education			<0.001	‡	
Illiterate	34 (0.5)	1.6 (0.6-4.1)	0.359		
>6 grades	293 (4.6)	1.4 (0.9-2.0)	0.110		
Elementary	702 (10.9)	1.2 (0.9-1.5)	0.268		
High school	1,637 (25.6)	1.1 (0.9-1.4)	0.291		
Pre-university	1,868 (20.2)	Ref	-		
Polytechnic	800 (12.5)	0.8 (0.6-1.1)	0.257		
University	904 (14.1)	0.9 (0.7-1.1)	0.328		
Other	20 (0.3)	†	0.966		
<6 years old	127 (1.9)	4.8 (3.2-7.2)	<0.001		
No response∗	14 (0.2)				

∗Individuals who did not respond were excluded from regression analysis

†An odds ratio could not be calculated because there were zero cases in that category

‡Variable was excluded from multivariable regression analysis due to high correlation between one or more categories of the variable and categories of the gender and/or age variables

CI=Confidence interval; OR=Odds ratio; Ref=Reference group

### Response rate and representativeness of respondents

Of the 6,576 households contacted, 6,399 (97.3%) interviews were completed. The distribution of the demographic characteristics of the survey respondents is presented in Table 2. When compared the distribution in the population (Table 2), females were generally greatly over-represented, with the exception of CH during the dry season. Children aged 0-12 year(s) and teens aged 13-17 years were largely under-represented, especially during the dry season. Older adults aged 55-64 years and seniors aged 65+ years were over-represented in SC, and seniors were over-represented in CF and CH during the dry and the rainy season respectively. The number of respondents varied among the sentinel sites but was consistent between seasons within a site; this was expected because calculations of our sample size took into account the population of each municipality.

**Table 2.** Distribution (%) of demographic characteristics of the population in three sentinel sites in Cuba based on 2004 Census data and respondents in a burden-of-illness survey

Variable	Overall			Cienfuegos			Centro Habana			Santiago de Cuba		
	Population	Dry	Rainy	Population	Dry	Rainy	Population	Dry	Rainy	Population	Dry	Rainy
Number of population	816,634			164,180			157,539			494,915		
Number of respondents		3,187	3,212		548	548		1,084	1,109		1,555	1,555
Gender (%)												
Male		33.4	30.5	49.6	27.7	28.3	47.0	44.2	35.4	48.8	27.8	27.9
Female		66.6	69.5	50.4	72.3	71.7	53.0	55.8	64.7	51.2	72.2	72.1
Age-group (years) (%)												
0-12		1.4	4.9	16.5	1.1	3.1	13.7	2.5	6.8	16.3	0.7	4.2
13-17		2.6	2.8	7.7	0.9	2.2	6.4	4.4	4.1	7.7	2.0	2.1
18-24		9.8	7.8	9.4	12.0	8.0	8.1	11.4	8.2	10.0	7.9	7.5
25-54		51.3	49.1	46.9	52.4	58.4	46.5	53.8	43.2	46.6	49.3	50.1
55-64		15.8	16.8	9.4	12.0	15.3	10.5	13.0	15.2	9.6	19.2	18.5
65+		19.0	18.5	10.1	21.5	12.9	14.7	14.9	22.5	9.8	20.9	17.6

### Magnitude and distribution of illness

The overall prevalence of acute gastrointestinal illness was 10.6% within the previous month (680 of 6,399 respondents); however, it varied significantly by sentinel site and by season (Table 1 and 3). The prevalence during the rainy season (12.2-19.2%) was consistently higher than the dry season (3.4-7.0%) in all the sites (Table 3).

**Table 3.** Proportion (reported as %) of respondents with acute gastrointestinal illness, for each gender and age-group, within each sentinel site and season in Cuba

Variable	Overall		Cienfuegos		Centro Habana		Santiago de Cuba	
	Dry	Rainy	Dry	Rainy	Dry	Rainy	Dry	Rainy
Total number of respondents	3,187	3,212	548	548	1,084	1,109	1,555	1,555
Number of ill respondents	150	530	21	96	76	135	53	299
Ill respondents (%)	4.7	16.5	3.8	17.5	7.0	12.2	3.4	19.2
Gender (%)								
Male	42.0	34.2	23.8	28.1	40.8	41.5	50.9	32.8
Female	58.0	65.9	76.2	71.9	59.2	58.5	49.1	67.2
Age-group (years) (%)								
0-12	5.3	10.2	4.8	4.2	3.9	11.1	7.5	11.7
13-17	5.3	4.9	0	1.0	5.3	12.6	7.5	2.7
18-24	10.7	7.5	0	6.2	14.5	11.9	9.4	6.0
25-54	51.3	45.5	66.7	54.2	52.6	32.6	43.4	48.5
55-64	7.3	15.1	9.5	19.8	6.6	9.6	7.5	16.0
65+	20.0	16.8	19.0	14.6	17.1	22.2	24.5	15.0

Male-specific risks of morbidity ranged from 3.3 to 6.5 and 14.3 to 63.2 cases per 100 respondents during the dry and the rainy season respectively (Table 4). Female-specific risks of morbidity ranged from 2.3 to 7.4 and 11.0 to 51.1 cases per 100 respondents during the dry and the rainy season respectively. Controlling for age, the risk of acute gastrointestinal illness was higher in males than in females (OR=2.9, 95% CI 1.6-5.1, p<0.001) in SC during the dry season (Table 5).

**Table 4.** Age- and gender-specific risks of morbidity (and confidence intervals) per 100 respondents for acute gastrointestinal illness in three sentinel sites in Cuba

Variable	Overall		Cienfuegos		Centro Habana		Santiago de Cuba	
	Dry	Rainy	Dry	Rainy	Dry	Rainy	Dry	Rainy
Gender								
Male	5.9 (4.6-7.5)	18.5 (15.9-21.3)	3.3 (1.2-7.3)	17.4 (11.7-24.9)	6.5 (4.5-9.1)	14.3 (11.5-19.4)	6.2 (4.2-8.9)	63.2 (51.6-76.7)
Female	4.1 (3.3-5.0)	15.6 (14.1-17.3)	4.0 (2.3-6.4)	17.6 (13.8-22.1)	7.4 (5.5-9.9)	11.0 (8.8-13.7)	2.3 (1.5-3.4)	51.1 (44.4-58.6)
Age-group (years)								
0-12	18.2 (8.4-34.5)	34.4 (26.1-44.5)	16.7 (0.8-82.2)	23.5 (7.5-56.8)	11.1 (2.8-30.2)	20.0 (11.6-32.3)	36.4 (11.6-87.7)	53.8 (38.1-74.1)
13-17	9.5 (4.4-18.1)	28.6 (19.1-41.3)	0	8.3 (0.4-41.1)	8.3 (1.5-11.5)	36.9 (22.3-57.9)	12.9 (4.1-31.1)	24.2 (11.3-46.0)
18-24	5.1 (3.0-8.2)	15.9 (11.5-21.5)	0	13.6 (5.5-28.4)	8.9 (4.7-15.5)	17.6 (10.4-27.9)	4.1 (1.5-9.0)	15.5 (9.5-24.1)
25-54	4.7 (3.7-5.9)	15.3 (13.4-17.3)	4.9 (2.8-7.9)	16.3 (12.3-21.1)	6.9 (4.9-9.3)	9.2 (6.8-12.2)	3.0 (1.9-4.4)	18.6 (15.8-21.8)
55-64	2.2 (1.1-3.9)	14.5 (11.8-18.3)	3.0 (0.5-10.0)	22.6 (14.0-34.7)	3.5 (1.3-7.9)	7.7 (4.3-12.8)	1.3 (0.4-3.2)	16.7 (12.4-21.9)
65+	4.9 (3.4-6.9)	14.9 (12.1-18.4)	3.4 (1.1-8.2)	19.7 (11.2-32.2)	8.0 (4.5-13.4)	12.0 (8.3-16.9)	3.9 (2.2-6.6)	16.4 (12.1-21.8)

**Table 5.** Multivariable logistic regression models examining gender and age as risk factors for acute gastrointestinal illness in three sentinel sites in Cuba during the dry and rainy seasons

Variable	Overall		Cienfuegos		Centro Habana		Santiago de Cuba	
	Dry (n=3,187)	Rainy (n=3,212)	Dry (n=548)	Rainy (n=548)	Dry (n=1,084)	Rainy (n=1,109)	Dry (n=1,555)	Rainy (n=1,555)
		Odds ratio (95% confidence interval)							
Gender								
Male	1.4 (1.0-2.0)∗	1.1 (0.9-1.4)	0.8 (0.3-2.2)	1.0 (0.6-1.7)	0.9 (0.5-1.4)	1.2 (0.8-1.8)	2.9 (1.6-5.1)∗	1.2 (0.9-1.6)
Female	Ref	Ref	Ref	Ref	Ref	Ref	Ref	Ref
Age-group (years)								
0-12	4.5 (2.0-10.0)∗	2.9 (2.0-4.1)∗	4.1 (0.4-37.9)	1.6 (0.5-5.0)	1.7 (0.5-5.9)	2.4 (1.3-4.6)∗	22.7 (6.0-86.2)∗	4.9 (2.9-8.3)∗
13-17	2.1 (1.0-4.6)∗	2.2 (1.4-3.5)∗	∗∗	0.5 (0.1-3.7)	1.2 (0.4-3.5)	5.7 (2.9-11.2)∗	4.4 (1.4-13.7)∗	1.4 (0.6-3.0)
18-24	1.1 (0.6-1.9)	1.0 (0.7-1.5)	∗∗	0.8 (0.3-2.0)	1.3 (0.7-2.7)	2.0 (1.1-3.8)∗	1.4 (0.5-3.8)	0.8 (0.5-1.4)
25-54	Ref	Ref	Ref	Ref	Ref	Ref	Ref	Ref
55-64	0.5 (0.2-0.9)	1.0 (0.7-1.3)	0.6 (0.1-2.7)	1.5 (0.8-2.7)	0.5 (0.2-1.3)	1.4 (0.8-2.3)	0.5 (0.2-1.3)	0.9 (0.6-1.3)
65+	1.1 (0.7-1.6)	1.0 (0.8-1.3)	0.7 (0.2-2.1)	1.2 (0.7-2.4)	1.2 (0.6-2.3)	1.4 (0.8-2.3)	1.3 (0.7-2.6)	0.9 (0.6-1.2)

∗Significant at p<0.05

∗∗An odds ratio could not be calculated because there were zero cases in that category

CI=Confidence interval; Ref=Reference group

The risk of acute gastrointestinal illness differed by sentinel site. During the rainy season, the risk was higher in SC (relative risk [RR]=1.7, 95% CI 1.4-2.1, p<0.001) and CF (RR=1.5, 95% CI 1.1-2.0, p=0.003) than in CH. The opposite was found in the dry season; the risk was lower in SC (RR=0.5, 95% CI 0.3-0.7, p<0.0001) and CF (RR=0.5, 95% CI 0.3-0.9, p=0.01) than in CH. After controlling for age, gender, and season, the overall risk was lower in CH (OR=0.7, 95% CI 0.6-1.0, p=0.003) than in SC (Table 1).

Risks of morbidity among children ranged from 11.1 to 36.4 and 20.0 to 53.8 cases per 100 respondents during the dry and the rainy season respectively (Table 4). Risks of morbidity among teens ranged from 0 to 12.9 and 8.3 to 36.9 cases per 100 respondents during the dry and the rainy season respectively. Age-specific risks (controlling for gender) are shown in Table 5; adults aged 25-54 years were used as the comparison group. Children, teens, and young adults, aged 18-24 years, living in CH during the rainy season, were 2.4, 5.7, and 2.0 times more likely to experience acute gastrointestinal illness respectively. During the rainy season in SC, children were 4.9 times more likely whereas, during the dry season in SC, children and teens were 22.7 and 4.4 times more likely to experience acute gastrointestinal illness respectively. No significant differences were identified in any age-groups in CF (both the seasons) or in CH during the dry season.

### Symptoms and severity, medical care, and overall burden of illness

The most common type of diarrhoea reported by cases was watery (71.7-85.7% of the cases), followed by mucoid (4.8-14.6%) and bloody (1.3-4.8%) diarrhoea; the proportion of each type was relatively consistent between seasons and sites (Table 6). Additional symptoms were common and included abdominal pain, fever, chills, nausea, vomiting, and concurrent respiratory symptoms. Abdominal pain was the most frequent (38.1-78.7% of the cases), followed by chills (15.1-44.7%). The duration of illness ranged from 1 to 9 day(s) and varied by site, with cases in CF reporting longer durations than CH or SC. The mean duration ranged from 1.1 to 2.6 days and was significantly longer in CF than the other sites in both the seasons. The maximum number of episodes in 24 hours ranged from 8 to 15. The mean number was between 4 and 5, although significant differences between sites and seasons were noted. In CH and SC, more than 80% of the cases were ill for one day only whereas, in CF, roughly two-thirds of the cases were ill for more than one day. Severity, as measured by hospitalizations, was low with only eight (0.01%) of the 680 cases for both the seasons and all the sentinel sites. A household member had diarrhoeal illness in the seven days prior to the respondent becoming ill in 3.9-30.1% of the cases. The proportion was consistently higher during the rainy season in all the sentinel sites.

**Table 6.** Characteristics of acute gastrointestinal illness in three sentinel sites in Cuba

Characteristics	Overall		Cienfuegos		Centro Habana		Santiago de Cuba	
	Dry	Rainy	Dry	Rainy	Dry	Rainy	Dry	Rainy
	(n=150)	(n=530)	(n=21)	(n=96)	(n=76)	(n=135)	(n=53)	(n=299)
Type of diarrhoea (%)								
Watery	76.0	77.4	85.7	80.2	76.3	74.8	71.7	77.6
Mucoid	9.3	10.8	4.8	14.6	10.5	9.6	9.4	10.0
Bloody	2.7	2.6	4.8	2.1	1.3	2.9	3.8	2.7
Watery and mucoid	3.3	4.9	0	1.0	5.3	8.2	1.9	4.7
Watery, mu coid, and bloody	0	0.2	0	0	0	0	0	0.3
Watery and bloody	0	0.9	0	0	0	1.5	0	1.0
Mucoid and bloody	0	0.4	0	0	0	0.7	0	0.3
No response	8.7	2.8	4.8	2.1	6.6	2.2	13.2	3.3
Additional symptoms∗ (%)								
Abdominal pain	40.7	56.1	38.1	78.7	42.1	51.5	39.6	49.2
Fever	18.7	21.5	19.0	10.6	15.8	20.4	22.6	24.7
Chills	32.0	21.4	28.6	26.6	44.7	27.3	15.1	16.4
Nausea	13.3	23.3	14.3	17.0	10.5	17.4	17.0	27.1
Vomiting	12.7	18.6	28.6	18.1	10.5	18.9	9.4	18.1
Respiratory problems	0.7	2.5	0.0	2.1	0.0	1.5	1.9	3.0
Duration (days) of illness								
Range	1-6	1-9	1-6	1-9	1-3	1-3	1-3	1-4
Median	1	1	2	2	1	1	1	1
Mean (SD)	1.3 (0.8)	1.4 (0.9)	2.2 (1.5)†,‡	2.6 (1.8)†‡	1.2 (0.5)	1.1 (0.3)	1.1 (0.4)	1.2 (0.5)
% of cases with 1-day illness	81.1	81.7	35.0	37.3	86.4	92.6	92.4	88.0
Number of episodes per 24 hours								
Maximum	12	15	8	15	9	15	12	9
Median	4	4	3.5	4	4	4	3	4
Mean (SD)	4.4 (1.7)	4.7 (1.9)	4.7 (1.7)	4.6 (2.4)§	4.5 (1.6)§	5.0 (2.1)§	4.2 (1.9)	4.5 (1.7)
Household members ill with diarrhoea in previous 7 days (%)								
Yes	9.3	29.4	19.0	26.0	3.9	30.4	13.2	30.1
No	68.7	63.6	76.2	67.7	55.3	57.8	84.9	64.9
Unknown	22.0	7.0	4.8	6.3	40.8	11.8	1.9	5.0

∗Figures might add up to >100% because cases might have experienced more than one additional symptom

†Within a season, the duration of illness was significantly different from Centro Habana (p<0.0001)

‡Within a season, the duration of illness was significantly different from Santiago de Cuba (p<0.0001)

§Within a season, the mean number of episodes per 24 hours was significantly different from Santiago de Cuba (p<0.01)

SD=Standard deviation

The proportion of cases who visited a physician was fairly consistent between sites and seasons, ranging from 17.1% to 38.1% (Table 7). The most common reasons for not seeking medical attention were self-medication (23.1-57.1%), the illness was not severe enough (22.2-53.9%), and use of traditional medicine (15.4-32.0%). In CH and SC, the most common reason was self-medication whereas, in CF, the most common reason was that the illness was not severe enough. One or more diagnostic tests were requested by a physician for 35.5-62.5% of the cases who sought medical attention. The proportion was consistently higher during the dry season in all the sites. The most common type of sample requested was stool. Of those seeking medical care, 33.3-53.9% were asked to submit a stool sample (Table 8). Most (≥80%) stool samples were tested for parasites whereas fewer (0-50%) were cultured for bacteria (Table 7). A high proportion (>72%) of the cases submitted a stool sample when one of them was requested; the lowest compliance was in CF (Table 8). One or more treatments were prescribed for most (>86%) cases who visited a physician, with the exception of cases (25%) living in CF during the dry season (Table 7). Rehydration salts and antidiarrhoeals were the most common treatments. Of the cases who visited a physician and were prescribed a treatment, 0-31.6% received an antibiotic; the proportion was consistently higher during the rainy season.

**Table 7.** Physician-visits, treatments prescribed, and diagnostic tests (reported as %) requested by cases of acute gastrointestinal illness in three sentinel sites in Cuba

Physician-visits, treatment, and tests	Overall		Cienfuegos		Centro Habana		Santiago de Cuba	
	Dry	Rainy	Dry	Rainy	Dry	Rainy	Dry	Rainy
Physician-visit	(n=150)	(n=530)	(n=21)	(n=96)	(n=76)	(n=135)	(n=53)	(n=299)
Yes (%)	24.7	28.1	38.1	22.9	17.1	23.0	30.2	32.1
No (%)	75.3	72.9	61.9	77.1	82.9	77.0	69.8	67.9
Reason for not seeking medical attention	(n=113)	(n=382)	(n=13)	(n=74)	(n=63)	(n=105)	(n=37)	(n=203)
Illness not significant (%)	28.3	28.3	53.9	40.5	22.2	25.7	29.7	25.1
Self-medicated (%)	47.8	39.3	23.1	24.3	57.1	48.6	40.5	39.9
Traditional medicine (%)	21.2	25.4	15.4	16.2	20.6	19.0	24.3	32.0
Other (%)	0.9	2.9	0	4.1	0	6.7	2.7	0.5
Unknown (%)	1.8	4.2	7.7	14.9	0	0	2.7	2.5
Physician requested diagnostic test(s)	(n=37)	(n=149)	(n=8)	(n=22)	(n=13)	(n=31)	(n=16)	(n=96)
Yes (%)	54.1	39.6	62.5	54.5	53.8	35.5	50.0	37.5
No (%)	45.9	60.4	37.5	45.5	46.2	64.5	50.0	62.5
Type of laboratory test ∗	(n=20)	(n=57)	(n=5)	(n=12)	(n=7)	(n=11)	(n=8)	(n=36)
Stool (bacteria) (%)	25.0	11.9	0	16. 7	14.3	9.1	50.0	11.1
Stool (parasite) (%)	90.0	86.4	80.0	100.0	100.0	90.9	87.5	83.3
Blood (%)	5.0	16.9	0	33.3	0	27.3	12.5	8.3
Other (%)	5.0	13.6	20.0	16. 7	0	0	0	16.7
Treatment prescribed	(n=37)	(n=149)	(n=8)	(n=22)	(n=13)	(n=31)	(n=16)	(n=96)
Yes (%)	83. 8	96.0	25.0	86.4	100.0	96.8	100.0	97.9
No (%)	16.2	4.0	75.0	13.6	0	3.2	0	2.1
Type of treatment∗	(n=31)	(n=143)	(n=2)	(n=19)	(n=13)	(n=30)	(n=16)	(n=94)
Rehydration salts (%)	45.2	77.6	50.0	52.6	30.8	83.3	56.3	80.8
Antidiarrhoeals (%)	48.4	27.3	50.0	26.3	61.5	16. 7	37.5	30.8
Antibiotics (%)	6.5	18.9	0	31.6	0	16.7	12.5	17.0
Traditional medicine (%)	19.4	24.5	0	23.1	15.4	13.3	25.0	29.8
Antipyretics (%)	6.5	8.4	0	0	0	3.3	12.5	11.7
Antiemetics (%)	0	6.0	0	0	0	0	0	8.5
Combination of above (%)	25.8	42.7	0	15.8	7.7	23.3	43.8	54.3

∗Figures might add up to >100% because cases might have had more than one type of test or treatment

**Table 8.** Burden of acute gastrointestinal illness for three sentinel sites in Cuba

Burden-of-illness characteristics	Overall		Cienfuegos		Centro Habana		Santiago de Cuba	
	Dry	Rainy	Dry	Rainy	Dry	Rainy	Dry	Rainy
No. (%) of cases	150 (4.7)	530 (16.5)	21 (3.8)	96 (17.5)	76 (7.0)	135 (12.2)	53 (3.4)	299 (19.2)
No. (%) of cases seeking a physician	37 (24.7)	149 (28.1)	8 (38.1)	22 (22.9)	13 (17.1)	31 (23.0)	16 (30.2)	96 (32.1)
No. (%) from whom a stool sample was requested	18 (48.6)	53 (35.6)	4 (50.0)	11 (50.0)	7 (53.9)	10 (35.5)	7 (43.8)	32 (33.3)
No. (%) who submitted a stool sample	16 (88.9)	46 (86.8)	3 (75.0)	8 (72.7)	6 (85.7)	9 (90.0)	7 (100.0)	20 (90.6)

## DISCUSSION

Although study design and case definitions vary among studies, the overall monthly prevalence of acute gastrointestinal illness in Cuba is comparable with the prevalence estimates from retrospective, population-based burden-of-illness surveys conducted in developed countries (1-9). Since our study, researchers have proposed a standard symptom-based case definition for acute gastrointestinal illness to facilitate comparisons between studies (16). Strong seasonal differences in prevalence were observed. The prevalence was consistently higher during the rainy season in all the sentinel sites. A similar seasonal effect was observed in Argentina, where the prevalence was three-fold higher in the high season of acute gastrointestinal illness than the low season in the seven-day recall period (Thomas MK *et al*. Personal communication, 2008). Seasonal variation in prevalence has also been reported in developed countries in temperate regions (3-9).

There is a strong link between foodborne and waterborne diarrhoeal illnesses and weather- and climate-related events. Heavy rainfall has been associated with a large number of outbreaks of waterborne diseases in Canada and the USA and also an increased incidence of diarrhoea in Fiji (18-20). The incidence of diarrhoea generally rises in the rainy season in developing countries, partly because of direct effects of temperature and rainfall on the growth and spread of pathogens (21). Substandard hygiene and sewage disposal, inadequate facilities for food storage and preparation, and lack of potable water are factors that might contribute to a high incidence of gastrointestinal illness. Overwhelmed sewage systems might be an important contributor to the increased prevalence of acute gastrointestinal illness during the rainy season in Cuba.

The selection of one sentinel site per pre-defined acute gastrointestinal illness risk group (based on prior morbidity surveys) provided an opportunity for contrasting factors that might explain the differences in the burden of illness. Within the rainy season, CH had a significantly lower risk than the other sites. Since it is a densely-populated urban centre, the lower risk might be related to the absence of livestock and, therefore, decreased exposure to water sources contaminated by agricultural run-off. After adjusting for the effects of age, gender, and season, individuals living in CH continued to have a lower overall risk than individuals in SC. Rural residents have been shown to have a higher risk of acute gastrointestinal illness than urban residents (9), although others have found either no association (5-7), or an increased risk associated with urban living (2). Additionally, CH has a slightly older population than the other sentinel sites (Table 3). Seniors have often been reported to be less likely to experience acute gastrointestinal illness than adults or children (2,3,5,7-9). The site-specific risk differences were not consistent across seasons. Thus, other social determinants, such as living and working conditions, urbanization, informal economy (food distribution), lifestyle (consumption modes), and education efficiency could play a role in acute gastrointestinal illness.

Age was a significant risk factor for acute gastrointestinal illness in our study. Similar to burden-of-illness surveys conducted in developed countries (2,4-9) and in developing countries (21-23), children had a higher overall risk than adults. Stratifying the data revealed some interesting findings. Children were significantly more likely to experience acute gastrointestinal illness than adults in SC in both the seasons and in CH during the rainy season. Risks of morbidity were particularly high among children in SC during the rainy season. Age-specific risk differences revealed that, for children during the rainy season, there was an increased risk of acute gastrointestinal illness due to living in SC (compared to living in CH). Teens also had a higher overall risk than adults, and similar to children, this age-group was more likely to experience acute gastrointestinal illness than adults in SC during the dry season and in CH during the rainy season. Interestingly, children and teens in CF did not have a significantly different risk than adults, likely because this municipality in particular benefited from several governmental development programmes in the last decade.

In SC, during the dry season, males were three times more likely to experience acute gastrointestinal illness than females. However, even after controlling for age, season, and sentinel site, males had a higher overall risk. In Argentina, although males did not have a significantly higher risk than females overall, all cases aged less than 15 years were males (Thomas MK *et al*. Personal communication, 2008). A brief exploration of the interaction between gender and age in SC during the dry season did not reveal significant results. Our findings are in contrast to most burden-of-illness studies in developed countries, in which the prevalence is higher in females than in males (3,4,6-9). Although the reason for this is unclear, it is possible that there are cultural differences with respect to gender-related exposures. Since males were under-represented in our sample, these results should be interpreted with some caution. Nonetheless, targeted interventions and further research are warranted to identify exposures that might be unique to males in the Cuban population.

Symptoms described by cases of acute gastrointestinal illness in Cuba were similar to cases in developed countries, although the severity, measured by duration of illness and number of stools, tended to be the same or less (2-6,8,9). The significantly longer duration in CF might be related to differences in the type or virulence of pathogens in that region.

The proportion of cases visiting a physician was generally higher than in most other countries (2,3,5,8,9,17). Approximately 15-30% of the cases stated that they did not visit a physician because they used traditional medicine. This is similar to one Canadian study, in which 21% self-treated using herbal remedies (8). Of the cases who visited a physician, the proportion of those who were asked to submit a stool sample was generally higher than in developed countries (1-5,8,9,18), although rates of compliance were similar (2,4,5,8,17). Most stool samples tested were examined for parasites whereas a few were tested for bacterial pathogens. It is difficult to determine whether this finding reflects the actual medical practices or laboratory capacity in Cuba, or the respondent's understanding of the tests performed. With the exception of cases residing in CF during the dry season, most cases were prescribed a treatment by their physicians. The proportion of the cases taking antidiarrhoeal medication and antibiotics was similar to other countries (2-5,8,9). Taken together, this represents a large economic burden to the Cuban healthcare system.

Retrospective studies of self-reported acute gastrointestinal illness can be subject to recall bias and over-estimation of prevalence (1). Although this is a potential limitation of our study, our estimates are comparable with similar retrospective studies. Selection bias is a major limitation of our study. Children and teens were under-represented, and older adults, seniors, and females were over-represented. Consequently, it might not be possible to extrapolate the results to the general Cuban population. Random selection of individuals and administering interviews on various days and time of day should increase the generalizability of results in future studies.

This is the first study characterizing the burden of illness associated with gastrointestinal disease in Cuba. Acute gastrointestinal illness represented a substantial burden of health, comparable with, or higher than, developed countries. The risk was significantly higher during the rainy season in children and teens, in males, and in the municipality of SC. Ideally, these high-risk groups should be considered when allocating resources for education, food safety, and infrastructure.

## ACKNOWLEDGEMENTS

The study was supported by the Canada Biennial Programming Budget Fund through the Pan American Health Organization (PAHO).

The authors gratefully acknowledge Dr. Eleni Galanis, Dr. Dominique Charron, and the WHO for their contribution to the conceptualization of the study; Dr. Roland Miyar for assisting with all communications as a representative of the PAHO in Cuba; the Canadian Urban Institute for assistance with logistics and organizing workshops; Jessica Dennis for initial database management and data review; Rob Meyers for creating the map of the study area; and the provincial epidemiologists in Cienfuegos, Ciudad de La Habana, and Santiago de Cuba for their time and contribution to the study.
